# The addition of avibactam renders piperacillin an effective treatment for *Mycobacterium abscessus* infection in an in vivo model

**DOI:** 10.1186/s13756-018-0448-4

**Published:** 2018-12-13

**Authors:** Michal Meir, Pablo Bifani, Daniel Barkan

**Affiliations:** 1 The Ruth Rappaport Children’s Hospital, Rambam Health Care Campus, Haifa, Israel; 2 Koret School of Veterinary Medicine, The Robert H. Smith Faculty of Agriculture, Food and Environment, The Hebrew University of Jerusalem, Rehovot, Israel; 3 Department of Microbiology and Immunology, Yong Loo Lin School of Medicine, National University of Singapore, Singapore, Singapore; 4 Singapore Immunology Network (SIgN), A*STAR, Singapore, Singapore; 5 Faculty of Agriculture, School of Veterinary Medicine, POB12,, 76100 Rehovot, Israel

**Keywords:** *Mycobacterium abscessus*, Combination treatment, Avibactam, Piperacillin, MIC

## Abstract

Treating *M. abscessus* infection is challenging due to the potent β-lactamase Bla_Mab_ (Beta-lactamase of *M. abscessus*_)_. Avibactam is a non-β-lactam, β-lactamase inhibitor shown to inhibit Bla_Mab_. We tested whether avibactem can render piperacillin effective against *M. Abscessus*. In-vitro, avibactam enhanced the activity of piperacillin by 16–32 fold, with no significant effect on meropenem. In an in-vivo *Galleria mellonella* model, meropenem and piperacillin/avibactam significantly decreased infection burden compared to untreated controls. Neither piperacillin nor avibactam alone had a significant effect.

## Introduction, results and discussion

Non tuberculous mycobacteria (NTMs) are emerging pathogens in patients with cystic fibrosis (CF), recently estimated to affect approximately 12% of patients in Western countries [1]. Of NTM pulmonary infections, *Mycobacterium abscessus* infection is considered especially concerning as it is associated with increased morbidity and mortality, and is a poor prognostic factor even following lung transplantation [2–4]. Treatment of *M. abscessus* infections is challenging due to antibiotic resistance and tolerance mechanisms. Despite prolonged courses of multiple antibiotic treatments, long-term clearance of *M. abscessus* from respiratory airway in patients with CF is rarely successful [1]. Multi-bacterial infections, specifically of *M. abscessus*, *Pseudomonas aeruginosa* and sometimes *Staphylococcus aureus* are especially challenging and difficult to treat. Non-CF patients also suffer from *M. abscessus* infections, many times related to chronic lung diseases, plastic surgery, foreign bodies and other clinical situations.

Most β-lactam antibiotics, bar carbapenems and cefoxitin, are ineffective against *M. abscessus*, as it harbors Bla_Mab_ [5], a potent β-lactamase able to degrade both β-lactams and β-lactamase inhibitors. Avibactam is a new, non-β-lactam, β-lactamase inhibitor, active against Bla_Mab_ [5]. Data from zebrafish suggests avibactam can enhance the activity of ampicillin against *M. abscessus*, but ampicillin is inactive against *P. aeruginosa*. No data exists on whether it can augment the efficacy of the antipseudomonal drug piperacillin, enabling a single agent use against both *Pseudomonas aeruginosa* and *M. abscessus*, a co-infection often found in patients with CF [2].

In this study, we aimed to evaluate the effect of piperacillin/avibactam against *Mycobacterium abscessus*, in vitro and in vivo, using our recently established infection model in *Galleria mellonella* larvae [6], Coupled with a luminescent *M. abscessus* mutant (mDB158) [6].

To test the susceptibility of *M. abscessus* to piperacillin/avibactam in vivo, we used mDB158 [6], treated by meropenem, piperacillin, or ampicillin, each one with and without the addition of avibactam. Bacterial growth was assessed by luminescence measurement. Using 96 well plates, 5*10^3^ CFU of mDB158 were cultured with serial 1:2 dilutions of meropenem (50 to 0 mg/L), piperacillin (800 to 0 mg/L) and ampicillin (200 to 0 mg/L), alone or with the addition of 4 mg/L of avibactam. Following 72 h of incubation at 37 °C, luminescence was measured using the SpectraMaxi3® microplate detection system. At 72 h, luminescence consistently increased by 10^3^ fold in wells without antibiotics. The luminescence minimal inhibitory concentration (lu-MIC) was thus defined as the concentration in which luminescence remained similar to baseline value or increased no more than 3 fold compared to baseline. As expected, avibactam did not enhance the antibacterial activity of meropenem, as carbapenems are not considerably degraded by the β-lactamase of *M. abscessus*. The lu-MIC of ampicillin was reduced approximately 16 fold when augmented with avibactam, showing activity at 3.125 mg/L of ampicillin. Most importantly – the activity of piperacillin was enhanced 16–32 fold when combined with avibactam, also showing substantial antibacterial activity at 3.125 mg/L. Avibactam alone did not inhibit *M. abscessus* growth.

**Fig. 1:**
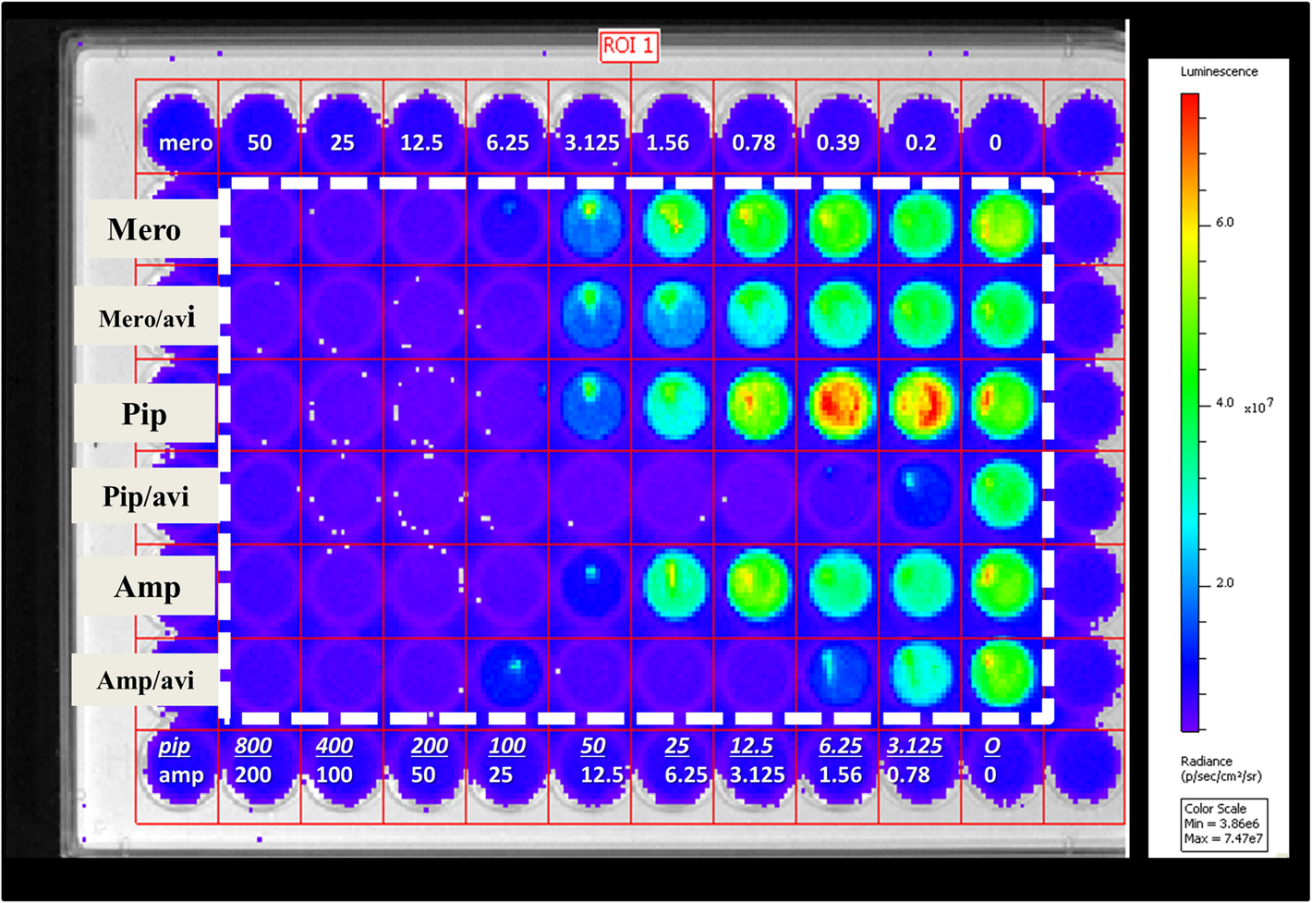
Avibactam lowers MIC of piperacillin for *M. abscessus*. A broth dilution assay was performed using 5*10^3^ CFU of a luminescent *M. abscessus* mutant with serial 1:2 dilutions of meropenem (50 to 0 mg/L), piperacillin (800 to 0 mg/L) and ampicillin (200 to 0 mg/L) alone or with the addition of 4 mg/L of avibactam. Image showing luminescence demonstrated by IVIS® following 72 h of incubation. Mero – meropenem, pip – piperacillin, amp – ampicillin, avi – avibactam. Dotted white line borders the test area. White numbers show mero/pip/amp concentrations

All the experiments/concentrations were done in triplicates, and repeated 3 times with similar results. For visual representation of these findings we analyzed a single 96-well plate with a single well for each concentration using the IVIS® imaging system (Fig. 1) with the above antibiotic combinations.

**Fig. 2:**
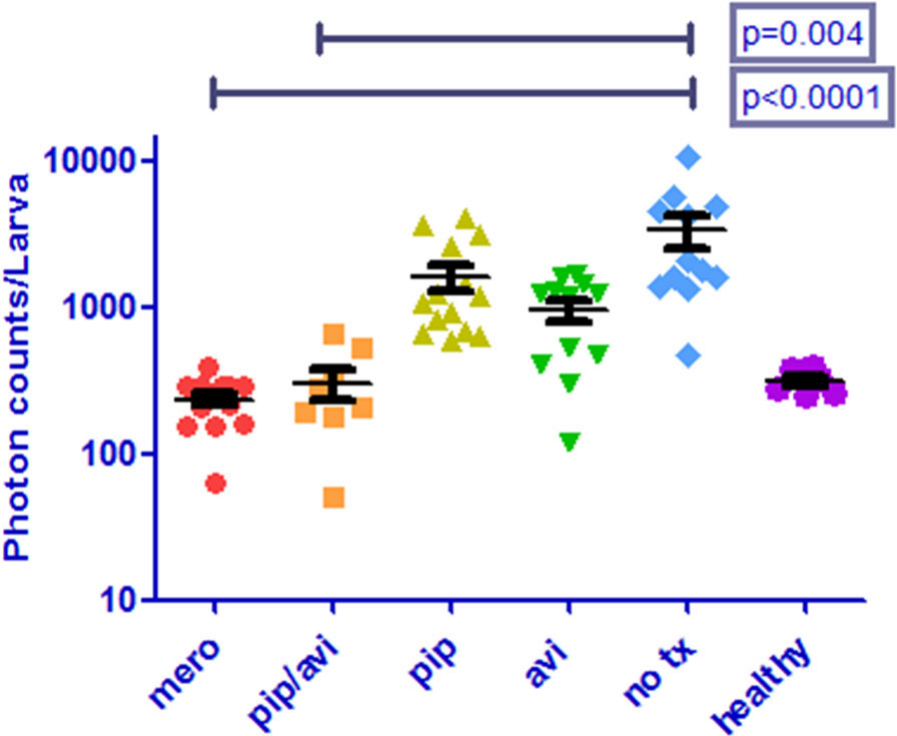
Piperacillin/avibactam is effective in treating *Mycobacterium abscessus* in a *Galleria mellonella* infection model. We inoculated 60 *G. mellonella* larvae with luminescent *M. abscessus* on day 0, and treated larvae with meropenem, piperacillin, avibactam alone, or piperacillin/avibactam on days 1 and 2. Using IVIS® imaging, we measured infection progression in live infected larvae on day 3. “Healthy” are un-infected larvae

To test if this combination is also effective in-vivo, we used our previously described *G. mellonella* larvae as a model of *M. abscessus* infection [6]. We inoculated 60 larvae with luminescent *M. abscessus* mDB158 on day 0 and kept them at 37 °C. On days 1 and 2 we treated larvae with 40 μg (200 mg/kg) meropenem, 100 μg (500 mg/kg) piperacillin, 0.2 μg (1 mg/kg) avibactam alone, or piperacillin combined with avibactam (100 μg/0.2 μg), approximating two daily doses of antibiotics. Using IVIS® Lumina Series III (Caliper LifeSciences), we measured infection progression in live infected larvae on day 3 (We previously showed RLU correlates well with CFU – [6]). Larvae treated with either meropenem or piperacillin/avibactam had a significantly lower infection burden compared to untreated controls (*p* < 0.0001 and *p* = 0.004 respectively). Piperacillin and avibactam alone had no significant inhibitory effect (Fig. 2). A second experiment with only one injection of antibiotics on day 1 showed similar results.

It is well established that pulmonary infection with *M. abscessus* is a poor prognostic factor for patients with CF, independently associated with a progressive decline in lung function [2–4, 7]. Treatment necessitates prolonged multi-drug regimens including a carbapenem backbone [1]. Use of a narrower spectrum β-lactam backbone has so far been hindered due to the *M. abscessus* potent β-lactamase Bla_Mab_ [5]. Avibactam was recently shown to inhibit Bla_Mab_, yet its role in treating this infection is unclear. Some in vitro data and in vivo zebrafish data demonstrated an ampicillin/avibactam combination to have an anti-mycobacterial effect [5]. Unfortunately, as patients with CF suffer from multi-bacterial infections including *Pseudomonas aeruginosa*, such a combination would not adequately target their pathogenic respiratory flora.

In our study, we showed piperacillin, an antipseudomonal β-lactam, to have a substantial effect against *M. abscessus* when augmented by avibactam. In vitro, 4 mg/L avibactam enhanced the activity of piperacillin 16–32 fold. This data suggests avibactam lowers the piperacillin MIC to a clinically-relevant range. In vivo, we also showed piperacillin/avibactam is able to treat *M. abscessus* infection in *G. mellonella* larvae, similarly to meropenem.

Our data suggests piperacillin/avibactam is a promising novel combination for patients with CF, targeting *M. abscessus*. Although the spectrum of piperacillin/avibactam is only mildly narrower than that of carbapenems (especially for *Acinetobacter*), it does spare the use of meropenem, slowing the development of carbapenem-specific resistance mechanisms. Use of piperacillin/avibactam may be especially useful for treating patients suffering from with *M. abscessus* and *P. aeruginosa* co-infections. Further in vivo studies are needed to establish efficacy, pharmacodynamics and pharmacokinetics of this combination.

## Abbreviations

BlaMab: β-lactamase of *M. abscessus*
CF: Cystic fibrosis
CFU: Colony forming unit
MIC: Minimal inhibitory concentration
NTM: Non tuberculous mycobacteria
RLU: Relative light unit (luminescence)
